# Integer programming-based method for grammar-based tree compression and its application to pattern extraction of glycan tree structures

**DOI:** 10.1186/1471-2105-11-S11-S4

**Published:** 2010-12-14

**Authors:** Yang Zhao, Morihiro Hayashida, Tatsuya Akutsu

**Affiliations:** 1 Bioinformatics Center, Institute for Chemical Research, Kyoto University, Gokasho, Uji, Kyoto, 611-0011, Japan

## Abstract

**Background:**

A bisection-type algorithm for the grammar-based compression of tree-structured data has been proposed recently. In this framework, an elementary ordered-tree grammar (EOTG) and an elementary unordered-tree grammar (EUTG) were defined, and an approximation algorithm was proposed.

**Results:**

In this paper, we propose an integer programming-based method that finds the minimum context-free grammar (CFG) for a given string under the condition that at most two symbols appear on the right-hand side of each production rule. Next, we extend this method to find the minimum EOTG and EUTG grammars for given ordered and unordered trees, respectively. Then, we conduct computational experiments for the ordered and unordered artificial trees. Finally, we apply our methods to pattern extraction of glycan tree structures.

**Conclusions:**

We propose integer programming-based methods that find the minimum CFG, EOTG, and EUTG for given strings, ordered and unordered trees. Our proposed methods for trees are useful for extracting patterns of glycan tree structures.

## Background

Data compression is useful because it can help reduce the consumption of expensive resources such as hard disks. To date, many methods such as Huffman coding, arithmetic coding, etc. have been proposed to solve problems of data compression. Data compression is also useful for the analysis of biological data. Li et al. proposed the universal similarity metric (USM), and approximated the dissimilarity using compression sizes. They applied a compression algorithm to unaligned mitochondrial genomes, and obtained a phylogeny that was consistent with the commonly accepted one [1]. Similarly, protein tertiary structures and metabolic networks were compressed, and their similarities were measured [2,3]. Grammar-based compression, which is a typical data-compression method, seeks a small grammar to generate a given string, as it is well known that it is NP-hard to find the smallest context-free grammar (CFG). However, in recent years, several polynomial time algorithms have been proposed to approximate the smallest grammar for the input data within a factor of *O*(log(*n/m*)), where *n* and *m* are the sizes of the input data and the smallest grammar [4-6], respectively. These algorithms can be used to compress biological data such as DNA, RNA, and amino acid sequences. However, there exist a large amount of tree-structured biological data (e.g., glycan, etc.). Therefore, it is necessary to develop methods to compress tree-structured data. Recent approaches show that it is feasible to extend the grammar-based compression to the tree-structure data [7-9]. However, these methods neither output the minimum grammar, nor they achieve a guaranteed approximation ratio.

In this paper, we propose an integer programming (IP)-based method that finds the minimum CFG for a given string under the condition that at most two symbols appear on the right-hand side of each production rule. Next, we extend this method to find the minimum elementary ordered-tree grammar (EOTG) and elementary unordered-tree grammar (EUTG) for given ordered and unordered trees. To the best of our knowledge, these are the first methods that can find the minimum size grammars for strings, ordered trees, and unordered trees.

It is possible to compress ordered trees by transforming them into Euler strings [10], and by applying existing grammar-based string-compression algorithms to the strings. Such an approach may achieve better compression performances. However, there do not always exist tree grammars corresponding to string grammars derived by the approach. Our objective is not only to compress trees but also to extract features and patterns from input trees. Therefore, we need to develop methods for finding minimum tree grammars. The organization of the paper is as follows. First, we give an IP-based method for sequence data compression using CFG. Second, we review an *elementary ordered tree grammar* (EOTG) [10] for ordered rooted tree compression, and extend this IP-based method to the tree compression problem. Then, we also review an *elementary unordered tree grammar* (EUTG) [10] for unordered rooted tree compression, and extend the above mentioned IP-based method for unordered trees. Furthermore, we conduct some computational experiments, apply the proposed methods to glycan tree-structure data, and extract tree patterns from generated production rules of simple EUTGs. Finally, we conclude with future work.

## IP formulation for strings

### Minimum CFG problem

We use a simple context-free grammar (CFG) for string and text compression. CFG is defined as 4-tuple (Σ, Γ, *S*, Δ), where Σ is a set of terminal symbols (denoted by a lower-case letter), Γ is a set of nonterminal symbols (denoted by an upper-case letter), *S* is a start symbol in Γ, and Δ is a set of production rules. The *size* of CFG is defined as the total number of letters appearing on the RHSs (Right-Hand Sides) of production rules. Two CFGs *G*_1_ and *G*_2_ are said to be *equivalent* if *G*_1_ generates the same set of strings as *G*_2_ does. We only consider CFGs consisting of the following types of production rules:

• *A*→*a*,

• *A*→*BC*.

We call this CFG *a simple CFG*. We can show that any CFG of size *m* can be transformed into an equivalent, simple CFG of size 3*m*.

The smallest grammar problem is thus defined as the problem of finding the smallest grammar that generates a given string [4].

### Minimum CFG

**Input:** String *s* = *s*_1_*s*_2_…s_*n*_ and integer *m*.

**Output:** Simple CFG with *m* nonterminal symbols that generates *s* only.

### Transformation to IP

We use the number of nonterminal symbols *m* instead of the size of the grammar because the number of terminal symbols appearing in production rules of *A* → *a* is constant for the given string. In order to solve this minimum CFG problem, we propose an IP-based method as follows. We transform this problem into the following integer program, where *x*_1,*n*_ = 1 holds iff there exists a required CFG *G*.

Maximize *x*_1,*n*_

Subject to

(1) *x*_*i*,*i*_ = 1 for all *i*=1*,…,n*

(2)(3)(4)(5)

In the above equations, each variable of *x*_*i*,*j*_, *y*_*i*,*k*,*j*_, and *z_u_* takes either 0 or 1. Each *x*_*i*,*j*_ corresponds to substring *s*_*i*,*j*_ = s_*i*_s_*i*+1_… s_*j*_, and *x*_*i*,*j*_ = 1 iff there exists a nonterminal symbol *A*_*i*,*j*_ in *G* that generates *s_i,j_*. *y_i,k,j_* = 1 iff both of *x_i,k_* = 1 and *x*_*k*+__1_*_,j_* = 1 hold. It means that *s*_*i*,*j*_ can be generated by concatenating *s*_*i*,*k*_ and *s*_*k*+1,_*_j_* that are generated from nonterminal symbols *A*_*i*,*k*_ and *A*_*k*+1__,*j*,_ respectively. *z_u_* = 1 iff there exists a nonterminal symbol in *G* that generates a substring *u* of *s.* The meaning of each (in)equality is as follows:

(**1**) each *s*_*i*,*i*_ (= *s*_*i*_) must be generated,

(**2,3**) if *A_i,j_* appears in *G, s_i,j_* must be generated, that is, for at least some *k*, both of *s_i,k_* and *s*_*k*+1_*_,j_* must be generated and the production rule *A*_*i*,*j*_ → *A*_*i*,*k*_*A*_*k*+1,*j*_ must appear in *G*,

(**4**) *A_i,j_* and *A*_*i'*,*j'*_, are identified if both generate the same substring *u*, and

(**5**) the number of nonterminal symbols used in *G* must be *m*.

**Figure 1:**
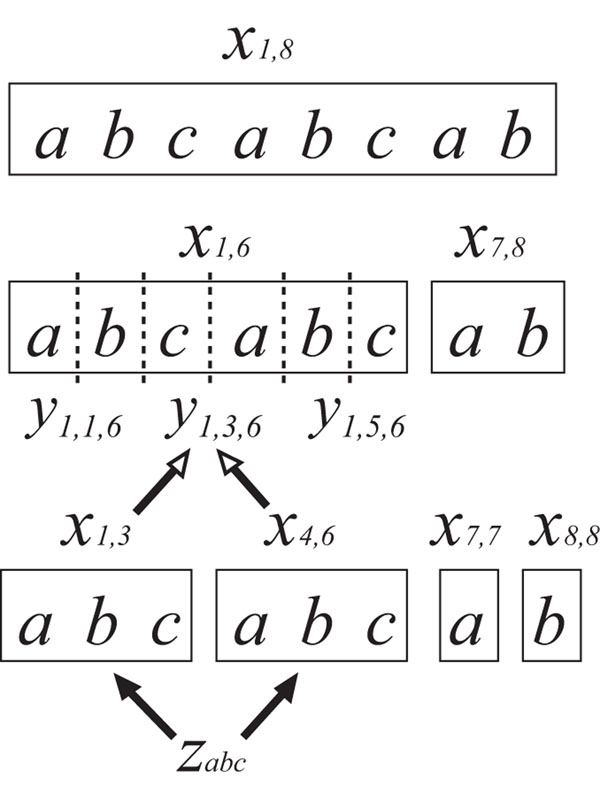
**Illustration of the minimum CFG for a string “abcabcab”** In the minimum CFG, the substring “abc” is generated from a nonterminal symbol *A_abc_*. Then, *z_abc_* = 1, *x*_1,3_ = 1, and *x*_4,6_ = 1. To generate “abcabc”, that is, *x*_1,6_ = 1, at least one variable of *y*_1,*k*,6_ (*k* = 1,⋯,5) must be 1, that is, the substring *s*_1,6_ must be divided into *s*_1,*k*_ and *s_k_*_+__1,6_. Here, *y*_1,3,6_ = 1 because *x*_1,3_ = *x*_4,6_ = 1. Then, the production rule *A_abcabc_ → A_abc_A_abc_* appears.

Figure 1 shows an example of the above IP formulation for the string “abcabcab”. For this example, the following grammar is constructed from a solution of IP:

A_1,8_ → A_1,6_A_7,8_,

A_1,6_ → A_1,3_A_4,6_,

A_1,3_ → A_1,2_A_3,3_,

A _4,6_ → A_4,5_A_6,6_,

A_7,8_ → A_7,7_A_8,8_,

….

On the other hand, we have *A_abcabcab_ = A*_1,8_, *A_abc_* = *A*_1,3_ = *A*_4,6_, etc. Therefore, we finally have:

*A_abcabcab_* → *A_abc_A_abc_A_ab_*,

*A_abc_* → *A_ab_A_c_*,

*A_ab_* → *A_a_A_b_*,

*A_a_* → *a*,

*A_b_* → *b*,

*A_c_* → *c*.

## IP formulation for ordered trees

### Minimum EOTG problem

We use a simple elementary ordered tree grammar (EOTG) [10] for rooted tree compression. In this grammar, a tree can contain a vertex called *a tag*. A tag indicates that another tree at the root can be attached to it. We assume that there is at most one tag in such a tree.

**Figure 2:**
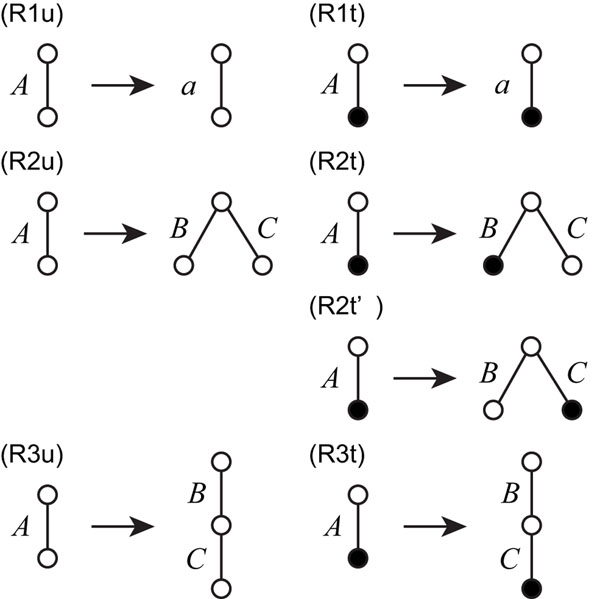
**Production rules of simple EOTG** A black circle denotes a tag.

A simple EOTG (SEOTG) is defined as 4-tuple (Σ,Γ, *S*, Δ), where Σ is a set of terminal symbols, Γ is a set of nonterminal symbols; each edge of the trees has either a terminal or a nonterminal symbol; *S* is a start symbol in Γ, and Δ is a set of production rules (R1u,t), (R2u,t,t’), and (R3u,t), as in Figure 2. (R1u) ((R1t)) denotes a rule when an untagged (tagged) edge of nonterminal symbol *A* is replaced with an untagged (tagged) edge of terminal symbol *a*. (R2u,t,t’) denotes a rule when an edge of a nonterminal symbol *A* is replaced with a tree that contains the upper endpoints of edges of nonterminal symbols *B* and *C* as the root, and the lower endpoints as two children. (R3u,t) denotes a rule when an edge of *A* is replaced with a tree in which the root is the upper endpoint of an edge of *B*, and the lower endpoint is the upper endpoint of an edge of *C*. We can show that any EOTG of size *m* can be transformed into an equivalent SEOTG of size 3*m*.

Within the class of SEOTGs, we can transform the minimum grammar problem into the IP. For this purpose, we define the minimum SEOTG problem as follows.

### Minimum SEOTG

**Input:** Rooted ordered tree *T*(*V, E*) and integer *m*, where *V* is a set of vertices and *E* is a set of labeled edges.

**Output:** Simple EOTG with *m* nonterminal symbols that generates only *T*.

## Transformation to IP

**Figure 3:**
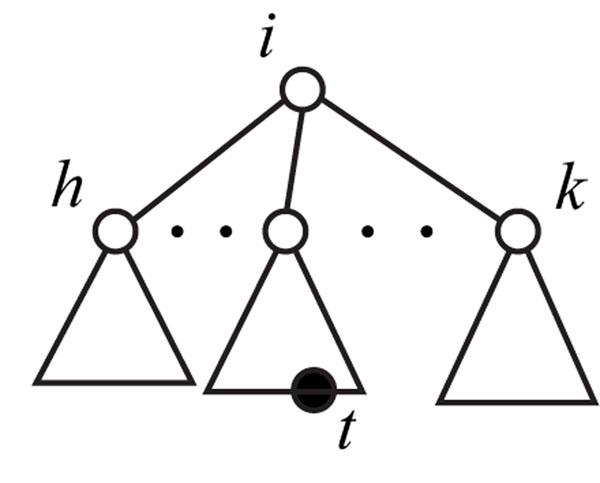
Example of ordered tree *T_i,t,h,k_*

A subtree of *T(V, E)* can be represented as a rooted ordered tree *T*_*i*,*t*,*h*,*k*_ with a root *i*, a tag *t*, a left-most child *h*, and a right-most child *k* of *i* (Figure 3), where the tree includes all the children of *i* between *h* and *k* in *T(V, E).* If a subtree does not contain any tag, we introduce ∈ that is not included in *V*, and represent it as *T_i,∈,h,k_*,. It is obvious from the production rules of simple EOTGs that it is sufficient to consider only such subtrees *T_i,t,h,k_* for *T(V, E)* because (R2u,t) denotes a rule to horizontally divide a tree into two trees at the root, and (R3u,t) denotes a rule to vertically divide a tree into two trees at an internal vertex that becomes a tag. Let *ch*(*i*) *=* (*lch*(*i*)*,…, rch*(*i*)) denote a sequence of all the children of *i* in *T*(*V, E*)*, lch*(*i*) is the left-most child, and *rch*(*i*) is the right-most child. Without loss of generality, we assume that *i*_1_*≤⋯≤ i_k_* for *ch*(*i*) = (*i*_1_, ⋯, *i_k_).* We suppose that the root of *T*(*V, E*) is 1. Then, this problem can be transformed into the following integer program, where *x*_1,*∈*,_*_lch_*_(1),_*_rch_*_(1)_ = 1 holds iff there exists a required EOTG *G*.

Maximize *x*_1,*∈*,_*_lch_*_(1),*rch*(1)_

**Figure 4:**
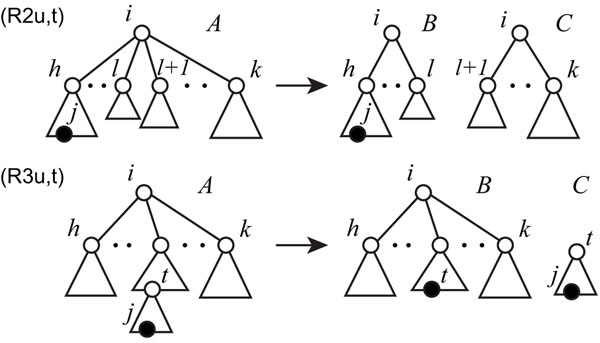
**Illustration for bisections of the tagged and untagged ordered trees for the production rules** Illustration for bisections of the tagged and untagged ordered trees for the production rules (R2u,t) and (R3u,t) of simple EOTG. *A*, *B*, and *C* correspond to nonterminal symbols in the production rules of Figure 2

Subject to

where *I*(*T_i,∈,h,k_*) denotes a set of internal vertices that are vertices (neither root or leaves) in *T_i,∈,h,k,_an*(*t*) denotes a set of ancestors of *i* (*i* ∉ *an*(*i*), and suppose *an*(*∈*) *=* ∅), and *es*(*T*) denotes the Euler string of the ordered tree *T* (for a tagged tree, the tagged edge with label *A* is transformed into *AxĀ*, where *x* is a special symbol representing the tag). It should be noted that if *es*(*T*) = *es(T'), T* is isomorphic to *T'*. In the above program, each variable of *x_i,j,h,k_*, , and *z_u_* takes either 0 or 1. Each *x_i,j,h,k_* corresponds to subtree *T_i,j,h,k,_* and *x_i,j,h,k_* = 1 iff there exists a nonterminal *A_i,j,h,k_* in *G* that generates *T_i,j,h,k_*. *z_u_* = 1 iff there exists a nonterminal symbol in *G* that generates subtree *u* of *T*(*V*, *E*). The meaning of each (in)equality is as follows (Figure 4):

**(1u,t)** each untagged (tagged) edge *T_i,∈,j,j_* (*T_i,j,j,j_*) must be generated,

**(2-3u,t)** if *A_i,j,h,k_* appears in *G* either for at least some *l*, *A_i,j,h,k_ → A_i,j,h,l_A_i,j,l_*_+1,_*_k_* ∈ *G*, or, for at least some *t, A_i,j,h,k_ → A_i,t,h,l_A_t,j,lch(t_*_),_*_rch_*_(_*_t_*_)_ ∈ *G* holds.  means that *T_i,j,h,k_* is horizontally divided at root *i* into the children {*s ∈ ch(i)|s ≤ l*} and {*s ∈ ch*(*i*)*|s > l*}.  means that *T*_*i*,*j*,*h*,*k*_ is vertically divided at *t* into two subtrees *T_i,t,h,k_* and *T_t,j,lch_*_(_*_t_*_),_*_rch_*_(_*_t_*_)_ (if *j* ≠ ∈, *t* must be in *an(j)*, otherwise, a divided tree would have two tags),

**(4)***A_i,j,h,k_* and *A_i_,_,j_,_h_,_k'_* are identified if both generate the same Euler string *u*, and

**(5)** the number of nonterminal symbols used in *G* must be *m*.

## IP formulation for unordered trees

**Figure 5:**
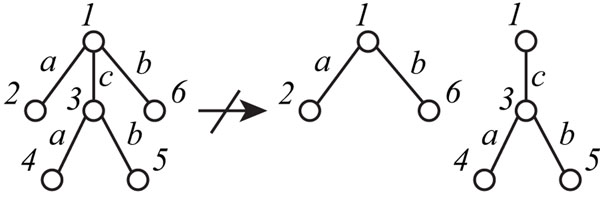
**Example of an ordered tree that is not well compressed** The left ordered tree cannot be divided into the two right trees. However, this is possible if the left tree is an unordered tree.

In some cases, a given ordered tree is not well compressed. Figure 5 shows an example of such a tree *T*(*V*,*E*), where edges (1, 2),(1, 3),(1, 6),(3, 4), and (3, 5) ∈ *E* are labeled with *a,c,b,a*, and *b*, respectively. A subtree *T*_3_*_,∈,_*_4,5_ is the same as a subtree with root *i.* However, we cannot divide the tree into such a subtree and the remaining part, as in the figure, according to production rules in EOTGs. Therefore, we need to extend the above IP for the ordered trees to that for the unordered trees. For this purpose, we extend the EOTG to a grammar for the unordered trees, called the elementary unordered tree grammar (EUTG) [10], and use a simple EUTG for rooted unordered tree compression.

**Figure 6:**
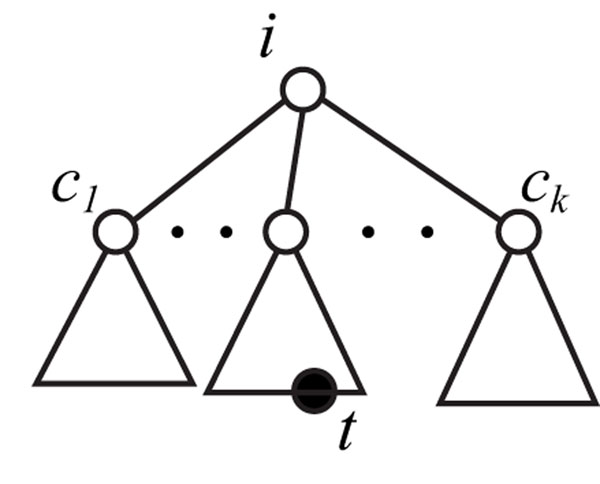
**Example of the unordered tree *T_i,t,C_*** Example of the unordered tree *T_i,t,_*_C_ with a set of children of *i, C* = {*c*1 *, ⋯, c_k_}*.

A simple EUTG (SEUTG) is defined as 4-tuple (Σ, Γ, *S*, Δ) in a similar way to EOTG. A set of production rules Δ is also the same as that of EOTG (Figure 2), except that trees appeared in the production rules are dealt as unordered trees. In other words, there is no sibling relationship between children *B* and *C* in the rules (R2u,t). Therefore, we must consider the subtrees of *T*(*V*, *E*) as *T_i,t,C_* (Figure 6), where *C* (≠ ∅) is a subset of the children of *i*. Although *ch*(*i*) is considered to be the sequence of children of *i* for the ordered trees, we allow it be a set of the children of *i* for the unordered trees.

Thus, within the class of SEUTGs, we define the minimum SEUTG problem as follows:

### Minimum SEUTG

**Input:** Rooted unordered tree *T(V, E)* and integer *m*, where *V* is a set of vertices and *E* is a set of labeled edges.

**Output:** Simple EUTG with *m* nonterminal symbols that generates only *T.*

We must identify unordered subtrees to count the number of nonterminal symbols *m*. For this purpose, we also use the Euler strings *es*(*T*) for the unordered trees *T* as in the minimum SEOTG. First, the unordered tree *T* is transformed into the ordered tree *T'* as follows. The children of each vertex in *T* are sorted by labels, and if it contains a tag, the tag is moved to the first of the children. Next, *es(T)* is calculated to be *es*(*T'*).

Thus, this problem is transformed into the following integer program, where *x*_1,∈_*_,ch_*_(1)_ = 1 holds iff there exists a required EUTG *G:*

Maximize *x*_1,*∈,ch*__(1)_

Subject to

## Computational experiments

We implemented the above mentioned IP-based methods for the ordered and unordered trees to perform some computational experiments. We used ILOG CPLEX (version 11.2, http://www.ilog.com/products/cplex/) to solve the integer programs. All of the computational experiments were conducted on a PC with a Xeon CPU 3.33 GHz and 10 GB RAM running under the LINUX OS. In our implementation, we first transformed the minimum grammar problem of the ordered and unordered trees into the integer programs. Next, we used ILOG CPLEX, and obtained the number of nonterminal symbols needed in the minimum grammars of this tree compression. Finally, as the results, the minimum grammars for the tree compression were constructed from the solution of the IP. We also tested the computational time of solving these integer programs. We performed experiments on both artificial data and the glycan tree-structure data, and compared our proposed methods with an existing method.

### Artificial data

**Figure 7:**
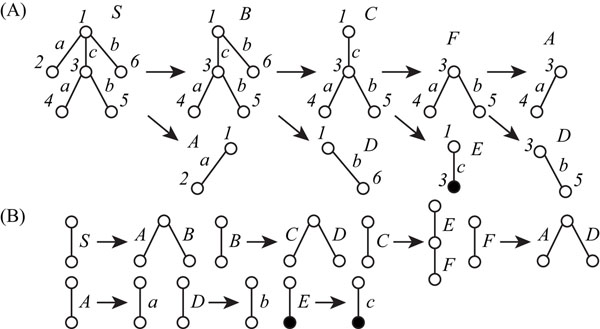
**Production rules generated by our IP-based method for the minimum SEOTG** (A) Derivation of production rules generated by our IP-based method for the minimum SEOTG. (B) Production rules generated by our IP-based method for the minimum SEOTG.

We chose the left tree *T* of Figure 5 in which edges labeled with “a” and “b” are connected to both endpoints of an edge labeled with “c”, and performed computational experiments, where the simple tree *T* was treated either as an ordered or an unordered tree. When *T* was regarded as an ordered tree, we generated the integer program with 13 nonterminal symbols for 9 horizontal and 4 vertical divisions. The number of nonterminal symbols needed in the minimum grammar of *T* is 7 because the number of production rules except (R1u,t) is 4 and the number of terminal symbols is 3. (Figure 7, in which nonterminal symbols, *S,A,⋯*, and *F* are used). The minimum grammar constructed from the solution of IP is as follows.

*T*_1,*∈*,2,6_ → *T*_1,*∈*,2,2_*T*_1,*∈*,3,6_ (1)

*T*_1,*∈*,3,6_ → *T*_1,*∈*,__3,3_*T*_1,*∈*,6,6_ (2)

*T*_1,*∈*,3,3_ → *T*_1,3,3,3_*T*_3,*∈*,4,5_ (3)

*T*_3,∈,4,5_ → *T*_3,∈,4,4_*T*_3,∈,5,5_. (4)

The production rules of this tree compression are also shown in Figure 7. The elapsed time to solve the IP was 0.014 s.

**Figure 8:**
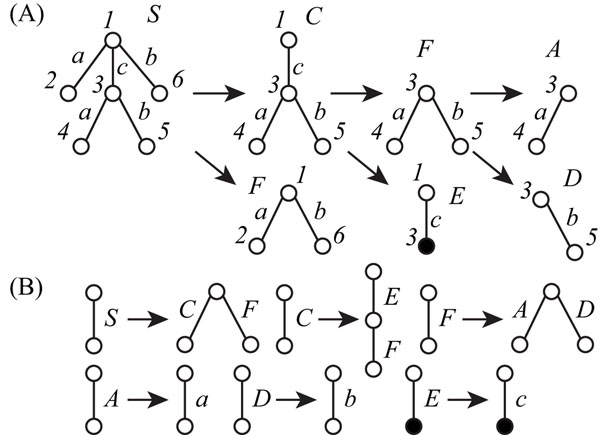
**Production rules generated by our IP-based method for the minimum SEUTG** (A) Derivation of production rules generated by our IP-based method for the minimum SEUTG. (B) Production rules generated by our IP-based method for the minimum SEUTG.

When *T* was regarded as an unordered tree, we generated the integer program with 14 nonterminal symbols for 12 horizontal and 4 vertical divisions. The minimum number of nonterminal symbols of *T* is 6 (Figure 8). The minimum grammar was constructed as follows.

*T*_1,*∈*,2,3,6_ → *T*_1,*∈*,3_*T*_1,*∈*,2,6_ (5)

*T*_1,*∈*,3_ → *T*_1,3,3_*T*_3,*∈*,4,5_ (6)

*T*_1,*∈*,2,6_ → *T*_1,*∈*,2_*T*_1,*∈*,6_ (7)

*T*_3,*∈*,4,5_ → *T*_3,*∈*,4_*T*_3,*∈*,5_. (8)

The production rules of this tree compression of *T* are also shown in Figure 8. The elapsed time to solve the IP was 0.016 s.

**Figure 9:**
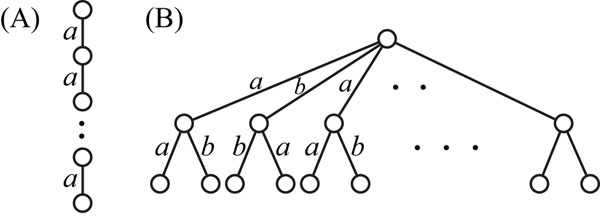
**Trees used in experiments for evaluation of our IP-based methods** (A) Trees having only vertices with degree at the most two. (B) Trees having vertices with degree more than two.

In addition to this simple example, we performed experiments for two types of trees with more vertices (Figure 9), where the number of vertices and degree was up to 61 and 20, respectively, and measured the elapsed times. Type A trees only contain vertices with the degree at most two and edges labeled with *a*, while Type B trees contain edges labeled with *a* and *b*, and the height is two. Table 1 shows the results on the elapsed time (seconds) to solve the minimum SEOTG and SEUTG problems by using CPLEX for the ordered and unordered trees of Type A and B with several sizes. *m* was the same as the minimum number of nonterminal symbols, except the case of Type A trees with 51 vertices. In these cases, CPLEX did not output the solution for *m* = 11 within 8 h. However, we were able to generate the production rules for *m* = 12, although 10 is the minimum number of nonterminal symbols. If we do not need the minimum grammar, then we can obtain the production rules faster than in the case of finding the minimum grammar. Furthermore, the results show that the elapsed time for an ordered Type A tree was almost the same as that for the corresponding unordered tree, and the time for an ordered Type B tree was shorter than that for the corresponding unordered tree. Even for the ordered tree with 61 vertices, the time was a few minutes. These results suggest that our proposed method is efficient for ordered trees. These results also suggest that the IP-based method for unordered trees should be used when sibling relationships do not have any meanings and the number of vertices and the maximum degree are not so large because the minimum SEUTG size is always smaller than the minimum SEOTG size. However, if the maximum degree is large and sufficient time is not given, the IP-based method for ordered trees should be used. It is because solving the minimum SEUTG problem for such trees may take too much time whereas the method for ordered trees is expected in many cases to provide a small grammar whose size is close to or the same as that of the smallest grammar obtained by the method for unordered trees.

**Table 1:** Results on the elapsed time (seconds) for ordered and unordered trees of type A and B

tree type	max degree	# vertices	ordered		unordered	

			*m*	time	*m*	time
A	2	11	7	0.021	7	0.019

A	2	31	10	302.74	10	329.20

A	2	41	10	8063.19	10	7730.64

A	2	51	12*	230.51	12*	233.44

B	3	7	9	0.011	8	0.010

B	6	19	11	0.185	10	1.108

B	8	25	11	1.404	10	26440.01

B	10	31	12	2.265	^-^	^-^

B	16	49	11	481.15	^-^	^-^

B	20	61	13	432.72	^-^	^-^

Results on the elapsed time (seconds) for ordered and unordered trees of type A and B (in Figure 9) with several sizes. m was the same as the minimum number of nonterminal symbols for each tree, except the case denoted by ’*’. ’-’ denotes that the solver took more than 8 hours.

### Glycan tree-structure data

**Figure 10:**
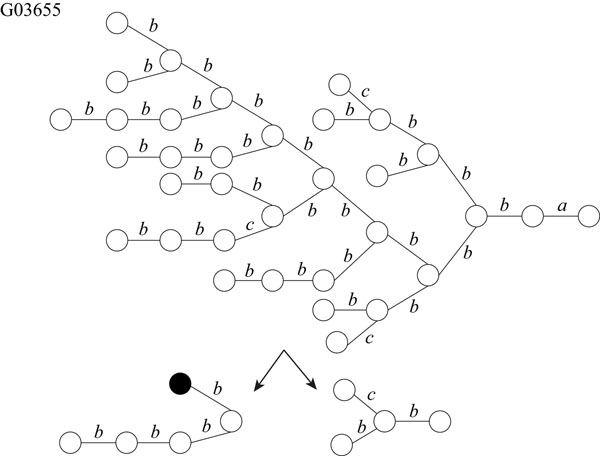
**Extracted patterns from glycan G03655** The label related with the lower endpoint is attached to each edge. Labels, *a*, *b*, and *c* denote GlcNAc, Man, and P, respectively.

**Figure 11:**
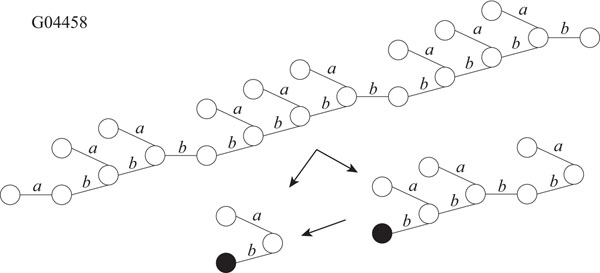
**Extracted patterns from glycan G04458** The label related with the lower endpoint is attached to each edge. Labels, *a*, and *b* denote Xyl, and Glc, respectively.

**Figure 12:**
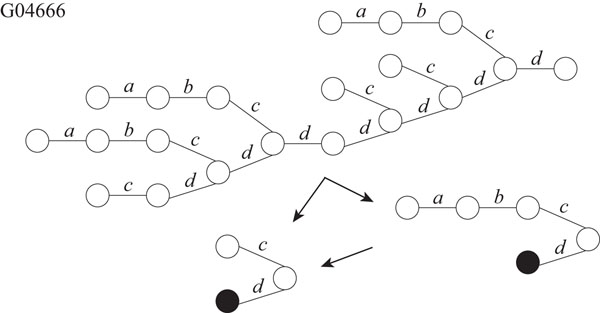
**Extracted patterns from glycan G04666** The label related with the lower endpoint is attached to each edge. Labels, *a, b, c*, and *d* denote LFuc, Gal, Xyl, and Glc, respectively.

**Figure 13:**
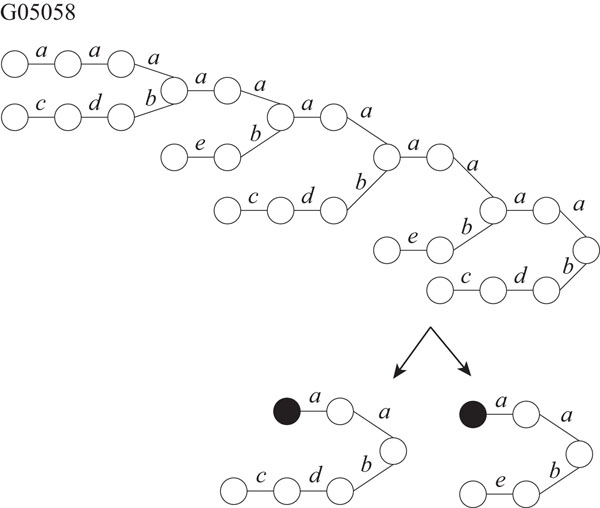
**Extracted patterns from glycan G05058** The label related with the lower endpoint is attached to each edge. Labels, *a, b*, *c*, *d*, and *e* denote Glc, Man6Ac, Man, GlcA, and 3-en-eryHexA, respectively.

It is known that glycans play important roles in a cell such as cellular adhesion and antigen-antibody reaction. Therefore, it is important to analyze structures of glycans. Hizukuri *et al.* extracted characteristic functional motifs of glycans, predicted a leukemia specific glycan motif, and confirmed by biological experiments that the *Agrocybe cylindracea* galectin specifically recognized human leukemic cells [11]. Thus, it is also important to find motifs and repeated patterns of glycans. We obtained twelve glycans, G02703, G03655, G03710, G04045, G04458, G04666, G04859, G05058, G05256, G05552, G06867, and G09054 as rooted trees from the KEGG Glycan database [12]. We labeled each edge with a lower-case letter corresponding to the type of sugar of the lower endpoint, because the edges are not labeled in the original data. For each glycan, the maximum degree, the number of vertices, and the number of distinct labels are shown in Table 2. Then, we applied our proposed IP-based method for SEUTG to each glycan as an unordered tree, and obtained the production rules. Figures 10, 11, 12, and 13 show extracted patterns from the production rules of G03655, G04458, G04666, and G05058. We can see from the result of the generated production rule that the tree of G03655 contains 2 of the same subtrees with 4 vertices and 3 of the same subtrees with 5 vertices, the tree of G04458 contains 2 of the same subtrees with 8 vertices and the subtree contains 3 of the same subtrees with 3 vertices, the tree of G04666 contains 3 of the same subtrees with 5 vertices and 2 of the same subtree with 3 vertices, and the tree of G05058 contains 3 of the same subtrees with 6 vertices and 2 of the same subtrees with 5 vertices. We were able to extract patterns similar to those of G03655, G04458, G04666, G05058 for the other glycans. The detailed derivation diagrams of production rules for the four glycans are available on our supplementary web site (http://sunflower.kuicr.kyoto-u.ac.jp/morihiro/treegram/).

**Table 2:** Statistics of glycans, G02703, G03655, G03710, G04045, G04458, G04666, G04859, G05058,G05256, G05552, G06867, and G09054, and results on the grammar size

glycan	max degree	# vertices	# distinct labels	Min SEOTG		Min SEUTG		TREE-BISECTION	
size	time	size	time	size	time

G02703	3	26	3	22	3.68	22	2.9	32	0.001

G03655	3	34	3	47	0.96	47	2.32	49	0.001

G03710	3	28	3	20	0.47	20	0.51	20	0.001

G04045	3	36	3	20	1.77	20	1.98	22	0.001

G04458	3	21	2	16	1.55	16	0.69	36	0.001

G04666	3	20	4	25	1.41	25	0.94	33	0.001

G04859	3	19	5	27	0.12	27	0.25	29	0.001

G05058	3	25	5	26	3.03	26	66.28	36	0.001

G05256	3	25	2	19	3.14	19	3.98	29	0.001

G05552	3	19	5	27	0.66	23	0.23	27	0.001

G06867	3	28	3	22	2.22	22	6.46	26	0.001

G09054	4	31	5	29	2.81	29	6.71	29	0.001

Statistics of glycans, G02703, G03655, G03710, G04045, G04458, G04666, G04859, G05058, G05256, G05552, G06867, and G09054, and results on the grammar size and the elapsed time (seconds) by our proposed IP-based methods for the minimum SEOTG and SEUTG problems, and TREE-BISECTION [10].

We compared the results of the grammar size for the minimum SEOTG and SEUTG by our methods with those of an existing method, TREE-BISECTION [10]. TREE-BISECTION repeatedly divides a given tree horizontally and vertically such that the size of a divided subtree is similar to that of another subtree until each subtree consists of an edge. It is known that TREE-BISECTION computes in polynomial time a simple EOTG of size *O*(*mn*^5/6^) [10], where *m* is the size of the minimum simple EOTG and *n* is the number of vertices of the given tree. Table 2 shows the results of the grammar size and the elapsed time by our proposed IP-based methods for the minimum SEOTG and SEUTG problems, and TREE-BISECTION. The minimum SEOTG size was the same as that of the minimum SEUTG for each glycan except G05552 because the tree contains vertices only with at most two children, and all subtrees of a vertex having three children are isomorphic. The size of the grammar generated by our methods was always smaller than or equal to that by TREE-BISECTION, and the ratio was 1.0 (G09054) to 2.25 (G04458). This result shows that our proposed method performs better with the compression ratio than TREE-BISECTION.

## Conclusions

We proposed integer programming-based methods for finding the minimum grammars to generate given strings, ordered trees, and unordered trees. By conducting computational experiments, we confirmed that our IP formulations work correctly. The results also show that our IP-based grammar compression is efficient for ordered trees, although some improvements are required for unordered trees.

We applied our proposed method to glycan tree-structure data, and extracted interesting patterns. Although these patterns were obtained from production rules generated for a single tree, we may be able to extract common patterns and rules from multiple glycans by extending our methods to find minimum grammars to generate given forests.

In this paper, we dealt with grammars for trees. However, real structured data often contain some cycles. Therefore, we are in the process of developing IP-based methods for more complex structured data.

## Competing interests

The authors declare that they have no competing interests.

## Authors contributions

TA gave the basic idea. YZ and MH developed and implemented the algorithms, and carried out the experiments. YZ, MH, and TA authored and approved the manuscript.
